# Prevalence of myopia and refractive parameters among children and adolescents in Hi-tech District of Chengdu City

**DOI:** 10.3389/fped.2024.1502660

**Published:** 2025-02-03

**Authors:** Xiaoqin Wang, Liuzhi Zeng, Yiping Xian, Mei Xin, Qingqing Shan, Weiye Li, Lixia Lv, Yifeng Liu, Rui Zhang, Kejian Song, Xixi Tian, Han Guo, Changjiang Yan, Chunyan Li, Xueni Luo, Honglin Luo, Ling Yang, Jun Luo, Zejun Chen

**Affiliations:** ^1^ Department of Ophthalmology, Chengdu First People's Hospital/Chengdu Integrated TCM & Western Medicine Hospital, Chengdu, Sichuan, China; ^2^ Department of Respiration, Chengdu First People's Hospital/Chengdu Integrated TCM & Western Medicine Hospital, Chengdu, Sichuan, China; ^3^ Development Department, Chengdu First People's Hospital/Chengdu Integrated TCM & Western Medicine Hospital, Chengdu, Sichuan, China; ^4^ High-tech Zone Healthcare Development Center, Chengdu, Sichuan, China; ^5^ High-tech Zone Education Development Center, Chengdu, Sichuan, China; ^6^ High-tech Zone Zhonghe Primary School, Chengdu, Sichuan, China; ^7^ Department of Ophthalmology, High-Tech Zone Zhonghe Community Health Service Center, Chengdu, Sichuan, China

**Keywords:** myopia, prevalence, spherical equivalent error, axial length, epidemiological

## Abstract

**Objective:**

To analyze the prevalence of myopia among children and adolescents in Chengdu from 2021 to 2023, providing insights for myopia prevention and control.

**Methods:**

This study was a school-based cross-sectional study in children and adolescents aged 3–18 years in Hi-tech District of Chengdu City. All the students underwent comprehensive ocular measurement, including uncorrected binocular visual acuity (VA), spherical equivalent error (SER) with noncycloplegic autorefraction, corneal radius (CR), and axial length (AL).

**Results:**

Over the three-year study period from 2021 to 2023, the overall prevalence of myopia was 38.15%, with annual rates of 38.74% in 2021, 38.67% in 2022, and 37.03% in 2023. The prevalence and severity of myopia increased significantly with age (*P* < 0.001). The prevalence was consistently higher among girls (*P* < 0.001) with 40.17% in 2021, 39.43% in 2022, and 38.33% in 2023 compared to 37.03%, 38.05% and 35.85% among boys in the years, respectively. The myopia prevalence increased with school level (*P* < 0.001). Mild myopia was the most common (24.47%), followed by moderate (10.77%) and severe myopia (2.91%). The mean SER were −1.72 ± 1.57 D in 2021, −1.32 ± 1.51 D in 2022, and −1.42 ± 1.36 D in 2023 (*P* < 0.001). AL was stable across most age groups, with mean AL across the overall sample of 23.80 ± 1.02 mm in 2021, 23.79 ± 1.04 mm in 2022, and 23.81 ± 1.04 mm in 2023.

**Conclusion:**

Myopic prevalence among children and adolescents in Hi-tech District of Chengdu City decreased from 2021 to 2023. The prevalence increased with age and school level, indicating a need for targeted interventions. Significant changes in spherical equivalent refraction and AL emphasize the importance of early intervention and regular monitoring of myopia for an effective management.

## Introduction

Myopia, also known as nearsightedness, has emerged as the predominant type of refractive error worldwide, significantly impacting visual health, particularly in Asian countries. By 2050, it is projected that nearly 50% of the global population will exhibit some degree of myopia, with high myopia affecting approximately 10% (1). The escalating prevalence of myopia poses a substantial public health challenge which is alarming due to the associated risk of severe ocular complications such as myopic macular degeneration, choroidal neovascularization, retinal detachment, and glaucoma, causing irreversible visual impairment (2).

Environmental and lifestyle factors, including increased near work activities and decreased outdoor exposure, have been implicated in the surge of myopia cases. Studies suggest that time spent outdoors may mitigate the onset and progression of myopia, likely through mechanisms involving retinal dopamine release stimulated by bright light (3). Moreover, the rise in myopia prevalence has been linked to socioeconomic factors, with a higher prevalence reported in urban and economically developed regions. This suggests a complex interplay of genetic, environmental, and lifestyle influences on myopia development and progression (4).

A higher rate of myopic progression among Chinese schoolchildren during the period of the COVID-19 pandemic was demonstrated and myopia progressed more rapidly during the period (5, 6). Despite extensive research, the dynamic trends of myopia in rapidly urbanizing regions remain under-explored. Moreover, there is a paucity of cross-sectional data on the prevalence of myopia among children and adolescents in this area. Therefore, we have assessed the prevalence of myopia among children and adolescents in Hi-tech District of Chengdu City in China, provide direction and theoretical basis for the prevention and control of myopia in children and adolescents and reduce the social and economic burden of the disease. This study aims to investigate the prevalence of myopia over a three-year period among school-aged children and adolescents in Chengdu. The result will be instrumental in developing effective myopia prevention strategies during the health policy planning.

## Methods

### Study design and subjects

This was a school-based, cross-sectional epidemiological study conducted between October 2021 and November 2023 in the Hi-tech District of Chengdu City, Sichuan Province, China. The study was conducted in accordance to the principles of the Declaration of Helsinki. All students and their guardians were informed about the objectives, contents, and significance of the study prior to enrolment and provided verbal agreement. Those diagnosed with eye diseases other than refractive errors, such as cataract, glaucoma, keratopathy, and retinal disease were excluded. Moreover, students wearing orthokeratology lenses were classified as myopic based on their pre-orthokeratology refractive error. The participating students were enrolled from all 228 schools including kindergartens, primary schools, junior high schools, and senior high schools within the district, where the compulsory education enrollment rate was 100%. Data collection was conducted annually from October to November to minimize variations in refractive status that might arise between the conclusion of the previous school year and the onset of the next.

### Examinations

Binocular uncorrected distance vision was tested using an international standard logarithmic visual acuity chart and a liquid crystal display (LCD) vision tester (Suowei). Visual acuity (VA) was recorded as decimals, with vision tested at a distance of 5 m. Non-cycloplegic refraction was measured using autorefractor (RKT-700A, NIDEK, Gamagori, Japan), with each eye measured at least three times. If any reading deviated by more than 0.50 diopters (D), the measurements were repeated until three consistent values were obtained which were then averaged for analysis. Ocular biometry parameters, including axial length (AL) and corneal radius (CR), were measured using non-contact partial coherence interferometry (SUOER SW-9000, China).

### Definitions

Myopia was defined as a spherical equivalent refraction (SER) ≤−0.75D without cycloplegia, and uncorrected visual acuity (UCVA) thresholds were set according to age: UCVA >0.3 logMAR for children aged 3, >0.2 logMAR for children aged 4 to 5, and >0 logMAR for those aged 6 or older (7, 8). Myopia was further categorized into three levels: mild myopia (<−0.5D to ≥−3.0D), moderate myopia (<−3.0D to ≥−6.0), and severe myopia (<−6.0D) (9).

### Data quality control

All procedures were performed by trained ophthalmologists, nurses, and technicians in line with standard operating protocols. Instruments were calibrated before use, and examiners underwent standardized training and assessment before the study. A detection error was defined as a discrepancy of more than ±1 line in visual acuity or ±0.50D in SER. If the error rate exceeded 5%, the testing data were retested (10). Data entry was meticulously double-checked to ensure accuracy; only data for the right eye was analyzed due to a high correlation between two eyes.

### Statistical analysis

The decimal vision was converted into minimum resolution angular logarithmic vision for statistical analysis. Data were analyzed using SPSS 20.0 (IBM SPSS, Chicago, IL, USA). Categorical data are presented as frequencies and percentages, while continuous data are given as means ± standard deviation or medians (range). Chi-squared or Fisher's exact tests were used to compare myopia prevalence across different subgroups. Univariate analysis of variance (ANOVA) was applied to compare refractive parameters across years. A *P*-value <0.05 was considered statistically significant.

## Results

### Patients' demographic characteristics

A total of 136,195, 146,342, and 165,460 students were initially enrolled in 2021, 2022, and 2023, respectively. Of the total, 128,706 in 2021, 140,558 in 2022, and 163,670 in 2023 were eligible for the final analysis with overall participation rate of 94.50%, 94.50%, 96.05%, and 98.92% for each respective year. Of the total sample, 66,617 were boys, accounting for 51.76%, and 62,089 were girls, accounting for 48.24% of the study population. In 2021, 22,982 students were kindergarten, accounting for 23.29%, age from 3 to 5 years. Primary school students were 6–12 years old with 66,194, accounting for 51.43%. 19,335 students in junior high school were 13–15 years old, accounting for 15.02%. Senior high school and vocational high school students were 16–18 years old with 13,195, accounting for 10.25%.

### Prevalence of myopia

Table 1 summarises the prevalence of myopia among genders, school type and age for the study years. Over the three-year study period from 2021 to 2023, the overall prevalence of myopia was 38.15%. Specifically, the prevalence of myopia was 38.74% in 2021, 38.67% in 2022, and 37.03% in 2023. Notably, the prevalence of myopia increased significantly with age (all *P* < 0.001). The prevalence of myopia was consistently higher among girls compared to boys in each year (*P* < 0.01). In 2021, the prevalence of myopia was 37.40% among boys and 40.17% among girls, with a similar trend observed in 2022 (38.05% in boys and 39.43% in girls) and 2023 (35.85% in boys and 38.33% in girls). The analysis of myopia prevalence across different school age segments revealed that the higher the school level, the higher the prevalence of myopia (all *P* < 0.001) (Table 1).

**Table 1:** Prevalence of myopia by student characteristics.

Variable		2021 year		2022 year		2023 year		*P*
		Myopia (*n*)	Myopia prevalence (%)	Myopia (*n*)	Myopia prevalence (%)	Myopia (*n*)	Myopia prevalence (%)	
Gender	Boy	24,913	37.40	27,584	38.05	30,357	35.85	0.01
	Girl	24,943	40.17	26,750	39.43	30,242	38.33	
School type	Kindergarten	954	3.18	2,797	7.08	1,140	3.33	<0.001
	Primary school	22,409	33.85	27,624	39.22	26,254	30.27	
	Junior high school	15,205	78.64	15,238	75.12	19,586	74.77	
	Senior high school	11,288	85.55	8,675	85.70	13,619	84.19	
Age (y)	3	264	3.64	377	5.81	360	4.23	<0.001
	4	385	3.3	479	5.68	425	3.52	
	5	247	2.38	441	4.77	226	1.87	
	6	1,154	9.15	1,500	9.78	921	5.15	
	7	2,245	16.7	1,947	14.00	1,904	11.19	
	8	3,565	31.51	3,664	26.26	3,590	24.86	
	9	4,837	41.44	4,352	37.59	5,520	40.07	
	10	4,977	55.63	6,398	51.41	6,300	53.12	
	11	5,117	64.56	5,524	57.87	7,438	61.18	
	12	5,073	72.15	5,739	63.79	7,047	68.31	
	13	5,381	78.69	4,873	66.77	6,732	75.61	
	14	4,954	83.82	5,512	78.78	5,877	80.45	
	15	3,770	84.91	4,853	81.05	5,101	83.49	
	16	3,894	85.78	4,290	86.25	4,784	84.6	
	17	3,425	85.89	3,652	85.91	3,841	84.51	
	18	568	80.23	733	81.72	533	89.43	
Total		49,856	38.74	54,334	38.67	60,599	37.03	<0.001

**Figure 1:**
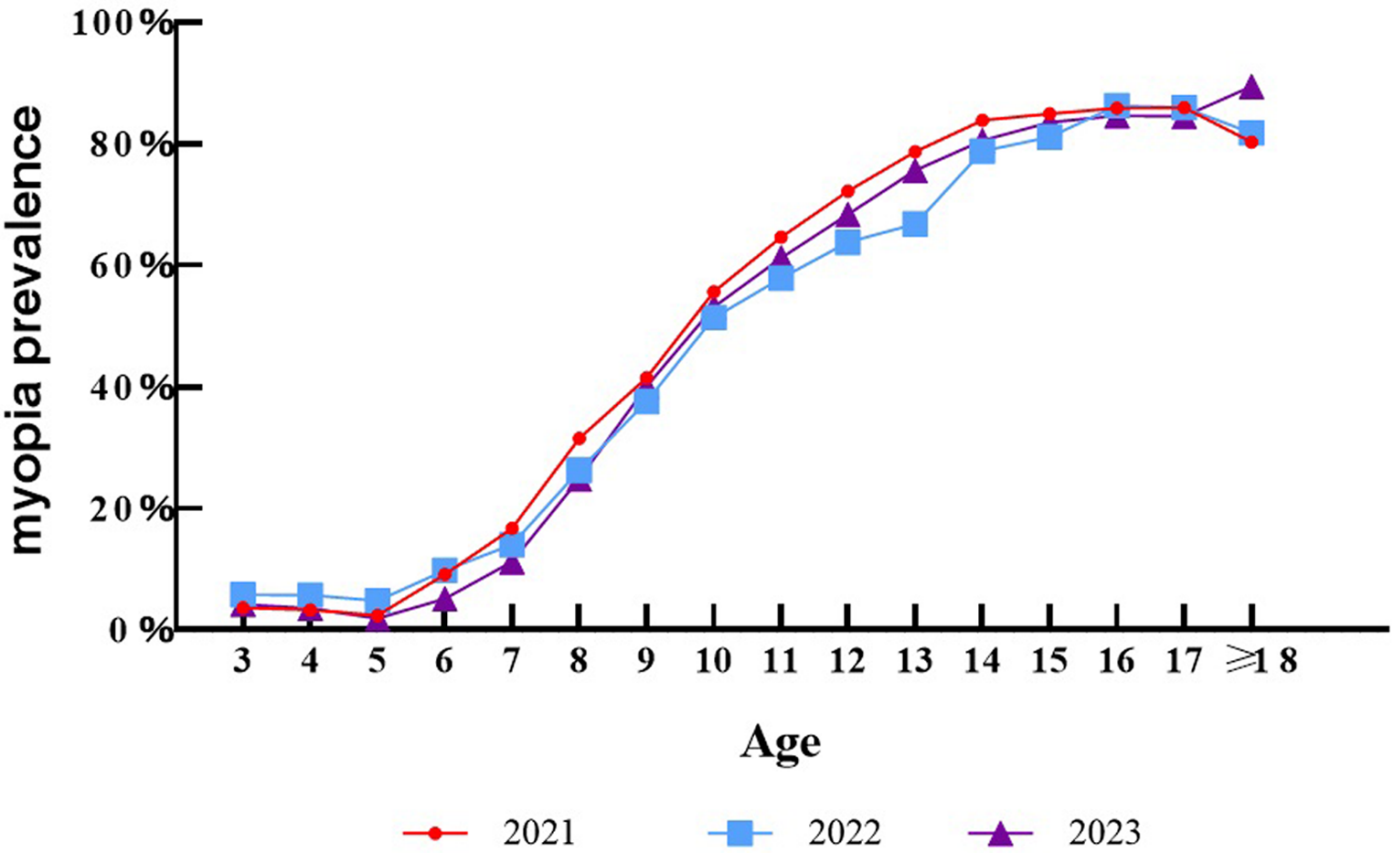
The distribution of myopia severity in each year.

**Figure 2:**
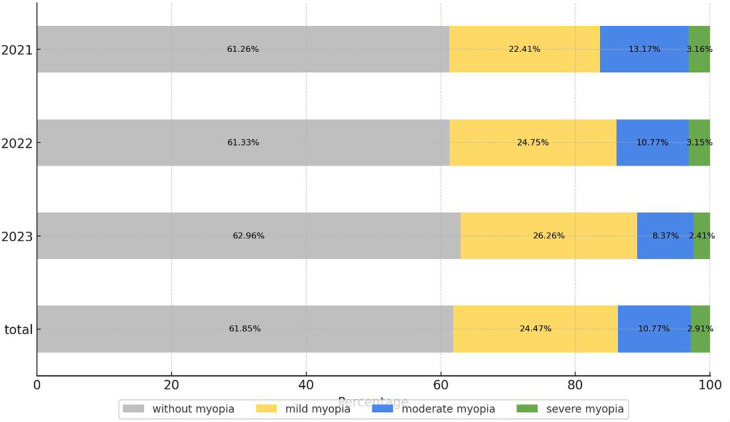
Myopia prevalences among children and adolescents at different ages in each year.

The detailed distribution of myopia severity in each year is depicted in Figure 1. Mild myopia constituted the largest proportion (24.47%), followed by moderate myopia (10.77%) and severe myopia (2.91%). There were statistically significant differences in the prevalence of myopia in different years, wherein the proportion of mild myopia in 2023 was significantly higher than that in 2021 and 2022, and there was no statistically significant difference in specific categories of myopia. As seen in Figure 2, the overall prevalence of myopia increased in a nearly linear fashion with age in each year, however, at the age of 14, the incidence of myopia was tending towards stability. The myopia rate of 3–5 years old was low and stable.

Majority of the children at the age 12 years of below had mild myopia, while the proportion of moderate myopia increased rapidly starting around 9 years old and stabilized after the age of 15. The prevalence of severe myopia began to increase at the age of 12 and gradually stabilized at the age of 16–18. With the increase of age, the degree of myopia in children and adolescents gradually increased, which is in line with the overall characteristics of myopia epidemic. The proportion of mild, moderate and severe myopia showed a step distribution, and the whole was dominated by mild and moderate myopia.

### Spherical equivalent refraction

The analysis of the SER revealed a variation over the three-year study period. Specifically, the overall mean SER was −1.72 ± 1.57D in 2021, −1.32 ± 1.51D in 2022 and −1.42 ± 1.36D in 2023 (*P* < 0.001). The SER of different age between 2021 and 2023 are shown in Table 2. The mean SER decreased significantly with age from 3 years-old to 18 years-old (*P* < 0.001). There were no significant differences in each year except ages 8–10 years and 13 years old. Children and adolescents in the age range of 3–6 years old showed farsightedness as a whole, 7 years old tended to be emmetropization, and 8–18 years old children and adolescents show myopia. This variation in SER may reflect changes in refractive error patterns among the student population over time.

**Table 2:** The SER of different age between 2021 and 2023.

Age (y)	2021 year		2022 year		2023 year		*P*
	*n*	SER (D)	*n*	SER (D)	*n*	SER (D)	
3	7,262	+0.21 ± 1.11	6,493	+0.24 ± 1.16	8,503	+0.13 ± 0.90	0.981
4	11,652	+0.17 ± 1.13	8,427	+0.28 ± 1.02	12,087	+0.16 ± 0.84	0.721
5	10,420	+0.12 ± 1.08	9,245	+0.34 ± 0.82	12,082	+0.21 ± 0.83	0.666
6	12,618	+0.10 ± 1.10	15,334	+0.30 ± 0.98	17,871	+0.23 ± 0.78	0.894
7	13,439	−0.21 ± 1.18	13,909	−0.26 ± 0.91	17,018	+0.01 ± 0.94	0.416
8	11,312	−0.52 ± 1.38	13,954	−0.03 ± 1.08	14,438	−0.32 ± 1.11	0.048*
9	11,674	−0.85 ± 1.61	11,579	−0.28 ± 1.24	13,776	−0.72 ± 1.32	0.001*
10	8,946	−1.37 ± 1.75	12,446	−0.61 ± 1.49	11,860	−1.15 ± 1.55	0.012*
11	7,927	−1.78 ± 1.99	9,546	−1.03 ± 1.77	12,157	−1.50 ± 1.71	0.095
12	7,032	−2.30 ± 2.06	8,997	−1.44 ± 1.85	10,316	−1.89 ± 1.86	0.244
13	6,838	−2.80 ± 2.22	7,298	−2.02 ± 2.17	8,903	−2.33 ± 1.98	0.041*
14	5,910	−3.36 ± 2.29	6,997	−2.34 ± 2.36	7,305	−2.76 ± 2.10	0.757
15	4,440	−3.65 ± 2.42	5,988	−3.12 ± 2.53	6,110	−2.97 ± 2.27	0.526
16	4,540	−3.89 ± 2.60	4,974	−3.81 ± 2.75	5,655	−3.23 ± 2.37	0.770
17	3,988	−3.93 ± 2.59	4,251	−3.72 ± 2.79	4,545	−3.42 ± 2.50	0.093
18	708	−3.54 ± 2.54	897	−3.58 ± 2.69	670	−3.16 ± 2.63	0.392

*n*, number; D, dioptres; SER, spherical equivalent error.

* Represents *P* < 0.05.

### Axial length and axial length/corneal radius

The results of the AL analyses across the age groups are detailed in the Table 3. The mean AL was 23.80 ± 1.02 mm in 2021, 23.79 ± 1.04 mm in 2022, and 23.81 ± 1.04 mm in 2023. From ages 5–17 years, there were no significant differences in AL changes between 2021 and 2023 (*P* > 0.05). Statistically significant differences in AL were primarily seen in the youngest (ages 3 and 4) and oldest (age 18) age groups. (*P* < 0.05). The AL appears to increase progressively with age, as expected, with the highest mean values observed in older age groups (15–17 years). The axial length/corneal radius (AL/CR) ratio were analyzed across different age groups from 2021 to 2023, as detailed in Table 4. The total AL/CR was 3.07 ± 0.13 in 2021, 3.08 ± 0.13 in 2022, and 3.07 ± 0.12 in 2023. The mean AL/CR showed a significant age-related increase (*P* < 0.05), while no significant annual variation was detected across the study period (*P* > 0.05).

**Table 3:** The axial length (AL) of different age between 2021 and 2023.

Age (y)	2021 year		2022 year		2023 year		*P*
	*n*	AL (mm)	*n*	AL (mm)	*n*	AL (mm)	
3	7,262	21.97 ± 0.68	6,493	21.93 ± 0.70	8,503	21.96 ± 0.71	0.009*
4	11,652	22.26 ± 0.68	8,427	22.18 ± 0.69	12,087	22.22 ± 0.68	0.028*
5	10,420	22.46 ± 0.70	9,245	22.43 ± 0.69	12,082	22.43 ± 0.68	0.536
6	12,618	22.68 ± 0.73	15,334	22.65 ± 0.72	17,871	22.67 ± 0.70	0.112
7	13,439	23.00 ± 0.77	13,909	22.98 ± 0.77	17,018	22.98 ± 0.76	0.307
8	11,312	23.35 ± 0.86	13,954	23.32 ± 0.83	14,438	23.32 ± 0.83	0.880
9	11,674	23.65 ± 0.92	11,579	23.65 ± 0.92	13,776	23.65 ± 0.87	0.648
10	8,946	23.94 ± 0.98	12,446	23.91 ± 0.99	11,860	23.94 ± 0.96	0.735
11	7,927	24.16 ± 1.03	9,546	24.12 ± 1.07	12,157	24.18 ± 1.00	0.596
12	7,032	24.37 ± 1.09	8,997	24.34 ± 1.09	10,316	24.41 ± 1.04	0.154
13	6,838	24.55 ± 1.13	7,298	24.58 ± 1.13	8,903	24.61 ± 1.08	0.536
14	5,910	24.78 ± 1.18	6,997	24.69 ± 1.18	7,305	24.81 ± 1.16	0.839
15	4,440	24.87 ± 1.24	5,988	24.87 ± 1.22	6,110	24.91 ± 1.17	0.317
16	4,540	24.95 ± 1.26	4,974	24.95 ± 1.23	5,655	24.98 ± 1.26	0.810
17	3,988	25.03 ± 1.28	4,251	25.03 ± 1.25	4,545	25.04 ± 1.26	0.307
18	708	24.78 ± 1.36	897	24.95 ± 1.38	670	24.85 ± 1.30	0.047*

*n*, number; mm, millimetres; AL, axial length.

* Represents *P* < 0.05.

**Table 4:** The AL/CR of different age between 2021 and 2023.

Age (y)	2021 year		2022 year		2023 year		*P*
	*n*	AL/CR	*n*	AL/CR	*n*	AL/CR	
3	7,262	2.83 ± 0.09	6,493	2.84 ± 0.09	8,503	2.83 ± 0.08	0.684
4	11,652	2.86 ± 0.09	8,427	2.87 ± 0.08	12,087	2.86 ± 0.08	0.551
5	10,420	2.89 ± 0.09	9,245	2.90 ± 0.08	12,082	2.88 ± 0.08	0.770
6	12,618	2.90 ± 0.10	15,334	2.93 ± 0.09	17,871	2.91 ± 0.08	0.822
7	13,439	2.93 ± 0.16	13,909	2.97 ± 0.09	17,018	2.94 ± 0.09	0.523
8	11,312	2.98 ± 0.86	13,954	3.00 ± 0.10	14,438	2.99 ± 0.09	0.962
9	11,674	3.01 ± 0.13	11,579	3.04 ± 0.11	13,776	3.02 ± 0.10	0.735
10	8,946	3.05 ± 0.12	12,446	3.07 ± 0.13	11,860	3.05 ± 0.11	0.757
11	7,927	3.08 ± 0.13	9,546	3.09 ± 0.15	12,157	3.07 ± 0.12	0.719
12	7,032	3.11 ± 0.13	8,997	3.12 ± 0.15	10,316	3.10 ± 0.12	0.564
13	6,838	3.13 ± 0.13	7,298	3.16 ± 0.15	8,903	3.12 ± 0.13	0.598
14	5,910	3.16 ± 0.14	6,997	3.17 ± 0.16	7,305	3.15 ± 0.14	0.953
15	4,440	3.17 ± 0.15	5,988	3.21 ± 0.16	6,110	3.16 ± 0.14	0.665
16	4,540	3.18 ± 0.15	4,974	3.22 ± 0.16	5,655	3.18 ± 0.15	0.882
17	3,988	3.19 ± 0.15	4,251	3.23 ± 0.17	4,545	3.19 ± 0.15	0.879
18	708	3.16 ± 0.16	897	3.21 ± 0.17	670	3.16 ± 0.16	0.755

*n*, number; AL, axial length; CR, corneal radius.

## Discussion

In this three-year epidemiological study conducted from 2021 to 2023, we comprehensively assessed the prevalence of myopia among children and adolescents residing in the Hi-tech District of Chengdu City. Our findings revealed a significantly higher prevalence of myopia in 2021 compared to the subsequent years, albeit with annual fluctuations. This study provides valuable insights into the evolving epidemiology of myopia and underscores the need for targeted interventions to address this public health concern. During 2021 and 2022, the COVID-19 pandemic was prevalent, leading to lockdowns in Chengdu and a subsequent reduction in students' outdoor activity time. However, in early 2023, with the easing of COVID-19 restrictions, there was an increase in outdoor activity time among students.

The present study observed a near-linear increase in the prevalence of myopia with age in each year, indicating a consistent pattern of myopia development across the school-aged population. These observations align with the results of previous domestic epidemiological investigations (11). Notably, the incidence of myopia tended to stabilize at age of 14 years, indicating a potential deceleration in the progression of myopia during the later adolescent years. This finding is consistent with prior research that associates myopia progression with the adolescent growth spurt. However, the leveling off of myopia prevalence after post-16 highlights the importance of implementing proactive myopia prevention and control measures during early adolescence to mitigate the rapid progression of myopia and reduce the risk of high myopia.

The significant annual changes in SER observed from 2021 to 2023 suggest notable shifts in refractive error patterns among school-aged children. The marked shift towards more negative SER values from 2022 to 2023 is particularly concerning, as it may be linked to increased online learning, reduced outdoor activities, and extended screen time. This trend reveals the profound impact of lifestyle changes on the visual health of youth, necessitating further investigation into these environmental factors and their role in refractive error development. During the outbreak of COVID-19, many environmental changes occurred including the reduced time outdoors and increase in screen time, which may contribute to the increase of myopia shift and elongation of axial length during the home quarantine (12). Future research should focus on devising targeted interventions to enhance visual health in students by mitigating modifiable lifestyle factors, such as promoting outdoor activities and regulating screen time (13).

Overall, the combination of SER and AL data provides insights into the refractive status and ocular growth patterns among school-aged children over time. As AL is a crucial indicator of myopia risk, accurate measurement and long-term monitoring of AL are essential for developing personalized myopia prevention strategies. Future research should focus on elucidating the physiological and pathological mechanisms underlying AL changes in these specific age groups. Understanding these factors is critical for the advancement of targeted interventions aimed at mitigating myopia progression in vulnerable populations (14). Given the strong correlation between the AL/CR ratio and SER, several studies propose that the AL/CR ratio may serve as a sensitive indicator for early myopia detection (15, 16). The combined use of UCVA and non-cycloplegic SER tests and the combined use of AL/CR and non-cycloplegic SER tests achieved optimal accuracy for myopia screening (17). In our study, the age-proportional increase in AL/CR aligns with previous studies associating higher AL/CR ratios with increased myopia risk during critical developmental years. These findings support the use of AL/CR as a reliable indicator to emphasize the importance of early screening in younger populations, particularly as the AL/CR ratio begins to approach critical thresholds predictive of myopia (15).

In this study, we acknowledge the potential bias introduced by using a noncycloplegic refraction, which tends to overestimate the magnitude of myopia. To mitigate this limitation, we have employed a rigorous definition of myopia that combines non-cycloplegic SER and UCVA measurements. Specifically, we defined myopia as a UCVA greater than 0.0 logMAR and SER of −0.75 D or less in right eye. This definition is intended to account for the non-cycloplegic nature of the autorefraction, which is known to overstate the prevalence of myopia among younger populations (18, 19). Given the concern about the overestimation of myopia prevalence due to non-cycloplegic refraction, researchers have explored alternative methods to improve accuracy (17). Thorn proposed that combining non-cycloplegic refraction with VA measurements can enhance the accuracy of myopia diagnosis compared to relying solely on non-cycloplegic refraction (20). We concur with this approach and have implemented it in our study. It is worth noting that for younger children aged 3–6 years, the criteria for myopia should be tailored to each age group, as the average visual acuity varies with age (17). Therefore, when applying our definition of myopia to this younger age group, it is essential to consider the normal range of VA for that specific age. By doing so, we can ensure a more precise and accurate assessment of myopia in children.

This comprehensive study accentuates a pivotal trend in the escalating prevalence of myopia based on school type, particularly as they progress through higher grade levels. The data reveals a stark increase, peaking at an alarming 85.70% in general high schools, which is attributed not only to the cumulative nature of myopia but potentially also to the augmentation of academic demands and the intensified use of the visual system as students ascend grade levels. Despite the introduction of policies aimed at alleviating the academic workload in various regions of China, and their diligent implementation by schools, the current burden on students remains substantial (21). Additionally, the phenomenon of parents adopting a “starting line” mentality, effectively shifting the reduced schoolwork pressure onto their children, has significantly augmented ocular strain, thereby contributing to the persistently high prevalence of myopia among children (22).

## Conclusions

This three-year long study revealed an overall myopia prevalence of 38.15% among children and adolescents in Hi-tech District of Chengdu City, with the highest rates observed among older students and girls. Myopic prevalence among children and adolescents was shown to decrease from 2021 to 2023. The prevalence increased with age and school level, highlighting the need for targeted myopia prevention and control strategies. Analysis of SER indicated significant changes in refractive error patterns, with myopia becoming prevalent from age 8 onwards. AL measurements showed consistent growth patterns across most age groups, though significant differences were noted at the youngest and oldest ages. These findings warrant the importance of early intervention and regular monitoring to address the rising trend of myopia and to support ocular health in school-aged populations.

## Data Availability

The original contributions presented in the study are included in the article/Supplementary Material, further inquiries can be directed to the corresponding author.
